# Ethyl Gallate: Promising Cytoprotective against HIV-1-Induced Cytopathy and Antiretroviral-Induced Cytotoxicity

**DOI:** 10.1155/2023/6727762

**Published:** 2023-07-12

**Authors:** C. Muddu Krishna, J. N. Kolla, Hari Babu Bollikolla, T. Sravan Kumar Reddy, S. Asha

**Affiliations:** ^1^Department of Biotechnology, VFSTR (Deemed to be University), Vadlamudi, Guntur, AP, India; ^2^Discovery Biology, Hetero Research Foundation, Hyderabad, Telangana, India; ^3^CZ-OPENSCREEN, Institute of Molecular Genetics of the Czech Academy of Sciences, Prague, Czech Republic; ^4^Department of Chemistry, Acharya Nagarjuna University, NNagar, Guntur-522510, AP, India

## Abstract

**Introduction:**

HIV-1 infection in cell culture is typically characterized by certain cytopathic effects such as vacuolization of cells and development of syncytia, which further lead to cell death. In addition, the majority of drugs during HIV treatment exhibit serious adverse effects in patients, apart from their beneficial role. During the screening of cytoprotective agents to protect the cells from HIV-1-associated cell death and also drug-associated toxicity, antioxidants from a natural source are assumed to be a choice. A well-known antioxidant, ethyl gallate (EG), was selected for cytoprotection studies which have already been proven as an anti-HIV agent.

**Objective:**

The main objective of the study was to explore the cytoprotective potential of EG against HIV-1-induced cytopathic effect and antiretroviral drug toxicity.

**Methods:**

DPPH free radical scavenging assay was performed with EG to find the effective concentration for antioxidant activity. HIV-1infection-associated cytopathic effects and further rescue by EG were studied in MT-2 lymphocytes by the microscopic method and XTT cytopathic assays. The cellular toxicity of different antiretroviral drugs in different cell lines and the consequent cytoprotective effectiveness of EG were investigated using an MTT cell viability assay.

**Results:**

Like ascorbic acid, EG exhibited promising antioxidant activity. HIV-1 infection of MT2 cells induces cell death often referred to as the cytopathic effect. In addition, the usage of antiretroviral drugs also causes severe adverse effects like cytotoxicity. In this context, EG was tested for its cytoprotective properties against HIV-1-induced cytopathic effect and drug-mediated cellular toxicity. EG reclaimed back the MT2 cells from HIV-1-induced cell death. Antiretroviral drugs, such as ritonavir, efavirinz, AZT, and nevirapine, were tested for their toxicity and induced more cell death at higher concentrations in different tissue models such as the liver (THLE-3), lung (AEpiCM), colorectal (HT-29), and brain (U87 MG). Pretreated cells with EG were rescued from the toxic doses of ART.

**Conclusion:**

EG was found to be exhibited cytoprotection not only from HIV-1-linked cell death but also from the chemotoxicity of antiretroviral drugs. Evidently, EG could be a cytoprotective supplement in the management of AIDS along with its enormous antioxidant benefits.

## 1. Introduction

Human immunodeficiency virus (HIV-1) infection in cell culture, especially lymphocytes, exhibits certain cytopathic manifestations, such as intracellular vacuolization and development of syncytia. These cytopathic appearances of cells consequently lead to cell death by budding and lysis at the cell membrane, further releasing a virus. The acute cellular infection of HIV-1 later progressed to chronic infection which causes effects like severe cell death [1]. Along with this, the treatment for HIV-1 infection and linked acquired immune deficiency syndrome (AIDS) was complex and involved multiple treatment regimens, but in many cases, the serious illness ensued from the continuing practice of antiretroviral drugs (ARTs). Certainly, growing evidence proves that the ARTs employed for HIV treatment have a toxic impact ensuing in many tissue pathologies. The highly active antiretroviral therapy (HAART) usually sets up a combination of multiple drugs like non-nucleoside reverse transcriptase inhibitor (NNRTI) in combination with nucleoside RT inhibitor (NRTI), an inhibitor of protease (PI) or integrase (IN) [2]. Many reports support that HAART moderates severe illness and is likely to avert infection [3]. The long term under HAART management is found to be harmful despite its benefits [4, 5]. The initial ART (antiretroviral treatment) regimens are NNRTIs or PIs with two NRTIs [6] but specified as neurotoxic and hepatotoxic as well as disturbing the reproductive system [7]. In certain cases, serious cardiovascular complications have been observed under ART treatment conditions [8]. The trademark NRTI has toxicity towards mitochondria and even exerts exterior myopathy or liver illnesses. The increase of free-radical species production and oxidative stress during HAART leads to many complications such as dementia, fungal and parasitic infections, lymphomas, neuropathy, and vacuolar myelopathy. Recently, many reports suggested the inhibitory activities of various phytochemicals against HIV-1 [9–11]. Distinct variances in vulnerability to antiretroviral toxicity persist to be exposed; on the other hand, numerous studies specify that genetic polymorphism may be convoluted, alongside ethnicity and gender. Endothelial dysfunction and autophagy, calcium imbalance, mitochondrial toxicity, and other many ART adversities are perceived in the gut, liver, kidneys, and major arteries [12]. Drug toxicity itself leftovers a primary reason for kidney disorders in many populations [13]. Hence, it may be beneficial to acquaint with a supplemental remedy that safeguards against drug-induced toxicity. Current drug development needs to focus not only on effective drugs but also need to curtail the side effects. Various natural products specifically betulinic acid (a triterpene), calanolides (coumarins), baicalin (a flavonoid), lithospermic acid (a polyphenolic), and polycitone A (an alkaloid) can be declared as anti-HIV molecules, and gallic acid and its derivatives are also reported for anti-HIV activities [14]. Natural compounds from food sources always pose to be safe and possess antioxidant activities. Gallic acid and its derivatives like methyl and ethyl gallate are known to be present in different plant sources including plant foods and act as natural antioxidants. The antioxidant nature of gallic acid is also involved in the regulation of viral replication, especially HCV in liver cells [15]. In our previous study, ethyl gallate (EG) was explored for anti-HIV activities and revealed inhibitory activity against different HIV-1 strains including drug-resistant isolates [16]. Supplementation of antioxidants along with antiviral drugs might help to reduce drug-associated toxicity and maintain the protection of the cells. In this scenario, EG was explored for cytoprotective activity which is widely present in many food sources. In the present study, EG was investigated for its cytoprotective properties against HIV-1-induced cytopathic effect and ART-mediated cytotoxicity.

## 2. Materials and Methods

### 2.1. Chemicals and Cell Culture Conditions

The test compound ethyl gallate; antiretroviral drugs such as ritonavir, efavirinz, AZT, and nevirapine; assay control doxorubicin, Eagle's minimum essential medium, trypsin/EDTA; and antibiotics such as penicillin/streptomycin, FBS (fetal bovine serum), and MTT (3-(4,5-dimethylthiazol-2-yl)-2,5-diphenyltetrazolium bromide) were purchased from Sigma-Aldrich (St. Louis, MO, USA).

The pulmonary alveolar epithelial cells (PAEpiC) were obtained from ScienCell Research Laboratories (Carlsbad, CA). The human colon adenocarcinoma cell line (HT29), nonmalignant hepatocyte cell line (THLE3), and human glioblastoma cell line (U87-MG) were procured from ATCC. HPAEpiC cells were maintained in Alveolar Epithelial Cell Medium (ScienCell, USA), and the remaining cell lines propagated in minimum essential medium with 5%FBS. Cells at a confluence of ∼90% were subjected to trypsinization and plated in 96-well cell culture microplates for the execution of cytotoxicity assays [17]. Experiments were carried out in duplicates and DMSO levels were limited to 1%.

### 2.2. Antioxidant Activity Assay

DPPH (1,1-diphenyl-2-picrylhydrazyl) free radical scavenging test was performed in 96-microplate according to the method of Ohnishi et al. [18]. Briefly, DPPH solution (80 *μ*l of 0.2 mg/mL) and EG (20 *μ*l) working solutions at different concentrations were added to the wells and mixed. Furthermore, sample mixtures were incubated for 30 min at 37°C under dark conditions. The multimode plate reader at 515 nm (Tecan INFINITE M1000) measured absorbance. The inhibitory effect was calculated by absorbance of the test sample and control; ascorbic acid was a positive control. All tests were run in duplicate.

### 2.3. HIV-1 Cytopathic Effect Inhibition Assay

MT2 cells (1 × 10^3^/well) were seeded in 96-well plates using RPMI complete medium, and cells were infected with HIV-1 viral strains such as 92HT 599 or IIIB (NIH AIDS Reagent Program, USA). Cells and viral titer were mixed in RPMI medium, kept for 1 h at 5% CO_2_ and 37°C, mixed to test compounds in 96-well microplates at variable concentrations, and further incubated for 96 h. After incubation, HIV-1 induced cytopathic effect and EG role was observed under a microscope; furthermore, cell viability was determined by XTT cell proliferation assay (Sigma, USA) in untreated cells, infected cells, and treated cells [19, 20].

### 2.4. Molecular Docking of EG against HIV-1 Capsid Protein

Docking simulations for the assessment of binding mode and interactions were carried out for the synthesized compounds. It was carried out through the iGEMDOCK software version 2.1 (https://gemdock.life.nctu.edu.tw/dock/). GEMDOCK stands for genetic evolutionary method for molecular docking. iGEMDOCK is a graphical automatic drug design system for docking, screening, and analysis. It is a program for computing ligand conformation and orientation relative to the active site of the protein. *In silico* docking simulation studies were performed to evaluate the molecular interactions of the ethyl gallate compound with the HIV-1 capsid protein (PDB: 6RWG). The 2D structure of the ligands was drawn through the BIOVIA Draw software and saved in the mol format. The ligand structures were optimized and minimized through the Avogadro software. The macromolecules were cleaned from water residues, and Gasteiger charges were added through Dock Prep in UCSF Chimera. The ligand interactions were visualized and analyzed through Discovery Visualizer (Biovia). A standard docking protocol was followed, and a stable docking method was selected [21]. Based on the scoring function, the best docking solutions were analyzed. The scoring function estimates the fitness by combining Van der Waal's hydrogen bonding and electrostatistic energies. Postdocking interaction profile analysis of best poses was conducted to determine the interactions between the ligand and the target protein.

### 2.5. Cytoprotection Assay

To evaluate the cytoprotection of cells from drug-related toxicity, the cells were preincubated with EG for 16 h and further incubated with respective antiviral drugs for 24 h. MTT cell viability assay was performed by following [22]. Moreover, the concentrations of EG that showed effective antioxidant activity was chosen for further experiments. The cells were plated in 96-well (20000/well) and incubated for 24 h. Subsequently, cells were pretreated with EG (100 *μ*M) for 16 h and further treated with antiviral drugs. Another set was maintained without supplementation of EG and further exposed to test arts. The plates were incubated for 24 h and further MTT assay was executed.

### 2.6. Statistical Analysis

For statistical analysis, a *t*-test (paired, 2-tailed) was performed between EG-treated cells and untreated control cells affected by the HIV-1 virus or drugs. A *p*value >0.05 is not reflected as statistically significant and is not denoted by any symbol. A *p*value <0.05 is resembled statistical significance and is specified with an asterisk (^*∗*^), a *p*value <0.01 is designated with a double asterisk (^*∗∗*^), and a triple asterisk (^*∗∗∗*^) is a representation of *p*value <0.001.

## 3. Results

### 3.1. Antioxidant Property of EG

**Figure 1:**
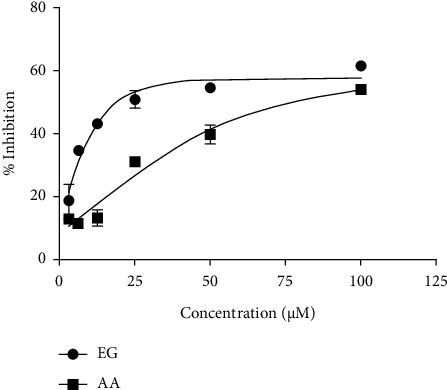
DPPH radical scavenging activity of EG: ethyl gallate and AA: ascorbic acid.

Although many studies revealed the antioxidant property of EG, the present study reconfirmed the antioxidant potency along with assay control of ascorbic acid.  Figure 1 exemplifies the DPPH scavenging activity of EG with ascorbic acid as an assay control. At a concentration of 100 *μ*M, EG attained superior inhibition such as positive control ascorbic acid. The inhibitory effect of EG on the DPPH radical was increased from 20% to 50% when the concentration ranged from 3 to 100 *μ*M. In the downward order, EG efficacy is better than that of ascorbic acid. The outcome specifies that EG employs a significant influence on scavenging free radicals. These results are in confirmation with Kalaivani et al. [23] who reported superior DPPH radical scavenging activity of EG isolated from leaves of *Acacia nilotica* than that of ascorbic acid.

### 3.2. HIV-1 Cytopathic Effect Inhibition Assay

**Figure 2:**
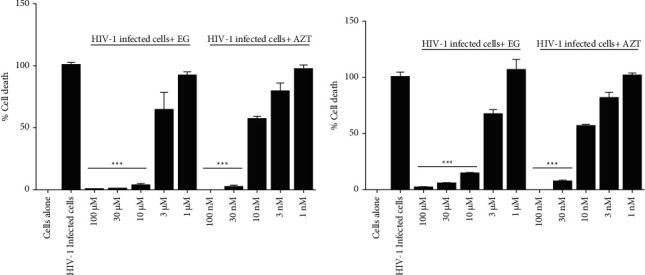
HIV-1 strain of (a) 92HT 599 and (b) IIIB cytopathic effect inhibitory activity of EG and AZT; EG: ethyl gallate; AZT: azidothymidine. Statistical analysis is performed by the paired *t*-test;^*∗*^shows *p*value <0.05, ^*∗∗*^shows *p*value <0.01, and ^*∗∗∗*^shows *p* value <0.001.

The HIV-1 strains 92HT 599 or IIIB infected the MT2 cells and developed cytopathic effects such as vacuolization of cells and the presence of syncytia. The syncytia induction in MT2 cells by HIV-1 strains was gradually inhibited with the increased concentration of EG. EG treatment of HIV-1 infected cells at the concentration of 100 *μ*M was completely retained back to normal phenotype and closely resembled the morphology of uninfected cells. The treatment of EG rescued the HIV-1 infected cells from the cytopathic effect in a dose-dependent manner (Figure 2). The tested viral strains caused cell death in the infected cells due to the viral-associated cytopathic effect. EG protected the cells from HIV-1 infection and rescued them from cell death. At 100 *μ*M concentration of EG, the viability was similar to noninfected cells (control) and rescued 100% from the infection of tested HIV-1 strains. The cytoprotection of EG (100 *μ*M) from HIV-1 infection was matched with the nontoxic concentration of the antiretroviral drug AZT (100 nM).

### 3.3. Molecular Docking of EG against HIV-1 Capsid Protein

**Figure 3:**
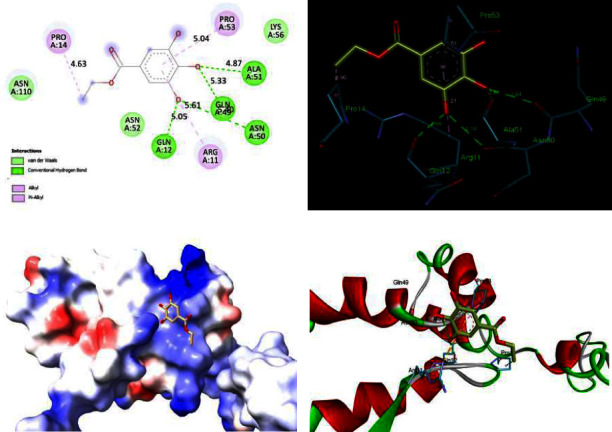
*In silico* analysis of EG binding towards HIV-1 capsid: (a) 2D plot of HIV-1 capsid protein-EG interactions, (b) H-bonds denoted as dashed green lines, (c) predicted binding and fitting of EG within the preferred binding cavity of HIV-1 capsid, and (d) binding mode of EG at the active site of HIV-1 capsid.

In our previous study, we proved the antiviral activity of ethyl gallate against different HIV-1 strains. Furthermore, we attempted molecular docking to find the *in silico* binding ability of ethyl gallate towards the protein of EG against HIV-1 capsid protein. Ethyl gallate interacts with amino acids such as GLN12, GLN49, ASN50, and ALA5 of HIV-1 capsid protein through H-bond and Alkyl, Pi-Alky interacts with ARG11, PRO14, and PRO53. The moiety OCH3 was mainly involved in the four H-bond interactions with the protein (Figure 3). These computational results are in agreement with the antiviral activity of ethyl gallate and are effective towards the inhibition of HIV-1 capsid maturation.

### 3.4. Cytoprotection of EG

**Figure 4:**
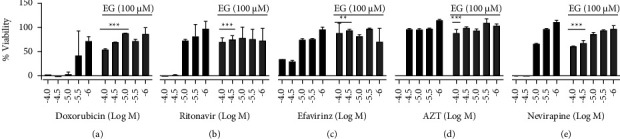
Cytoprotective effect of EG (100 *μ*M) on ART toxicities exerted in HPAEpic cells. (a) Doxorubicin (assay control); (b) ritonavir; (c) efavirinz; (d) AZT; (e) nivirapine. Statistical analysis is performed by the paired *t*-test;^*∗*^shows *p*value <0.05, ^*∗∗*^shows *p*value <0.01, and ^*∗∗∗*^shows *p*value <0.001.

HIV drugs have enriched over time, and severe adverse effects are less possible than they used to be. Nevertheless, HIV drugs still cause side effects. Some are minor, whereas others are more unadorned or even deadly. Adverse effects are prevailed as long as the drugs are used. Other treatments to interact with HIV drugs likely worsen the side effects. Other health circumstances can also mark the adverse effects of HIV drugs as worse. For these causes, when beginning any new drug, people need to focus on the supplement, which will compensate for the drug-related toxicity. In the current study, the cytoprotective influence of EG was studied on ART's drug-related toxicity. We selected four HIV drugs based on their severity of cytotoxicity along with an assay control doxorubicin. When cells are exposed to drugs alone, cells are susceptible to death when the concentration is increased. Two cancer cell lines (HT29 and U87-MG) and two normal cell lines (HPAEpic and THLE-3) of different tissue origin (colorectal, brain, lung, and liver, respectively) are utilized for the drug-induced cytotoxicity and further cytoprotection with EG.  Figure 4 depicts that the viability of lung cells (HPAEpic) was greatly reduced by approximately 100% when exposed to ritonavir and nevirapine at 30 *μ*M and above, and 70% of cell death was observed when it was treated with efavirenz at 30 *μ*M and above. But AZT exhibited effective cell death at 100 *μ*M. The pretreatment with EG (100 *μ*M) successfully improved the viability of the drug-assaulted cells; especially the rescue of cell death was very effective when the drug was at higher concentrations. It was evident that coincubation of 100 *μ*M EG was able to diminish the effects of toxic concentrations tested with HIV drugs as well as assay control doxorubicin on lung cells, while still sustaining greater than 50% viability at higher doses.

**Figure 5:**
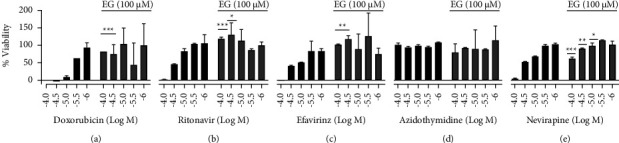
Cytoprotective effect of EG (100 *μ*M) on ART toxicities exerted in HT-29 cells. (a) Doxorubicin (assay control); (b) ritonavir; (c) efavirinz; (d) AZT; (e) nivirapine. Statistical analysis is performed by the paired *t*-test;^*∗*^shows *p* value <0.05, ^*∗∗*^shows *p* value <0.01, and ^*∗∗∗*^shows *p* value <0.001.

Cytoprotection of colorectal cells (HT-29) from the toxicity of HIV drugs was studied by using EG (Figure 5). The survival of HT-29 was greatly reduced by more than 90% when exposed to ritonavir, efavirenz, and nevirapine at 100 *μ*M, and up to 50% of cell death was observed when it was treated at 30 *μ*M. However, AZT was unable to display cell death until the concentration of 100 *μ*M. The pretreatment of cells with EG (100 *μ*M) successfully improved the viability from drug-associated toxicity. EG efficiently rescued the cells at a higher dose of toxicity of the HIV drugs. Treatment of 100 *μ*M EG was capable to reduce the toxic effects of HIV drugs as well as assay control doxorubicin on colorectal cells, and cytoprotection was evident at the higher doses of HIV drugs.

**Figure 6:**
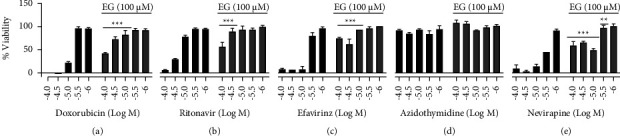
Cytoprotective effect of EG (100 µM) on ART toxicities exerted in THLE-3 cells. (a) Doxorubicin (assay control); (b) ritonavir; (c) efavirinz; (d) AZT; (e) nivirapine. Statistical analysis is performed by the paired *t*-test, ^*∗*^shows *p* value <0.05, ^*∗∗*^shows *p* value <0.01, and ^*∗∗∗*^shows *p* value <0.001.

**Figure 7:**
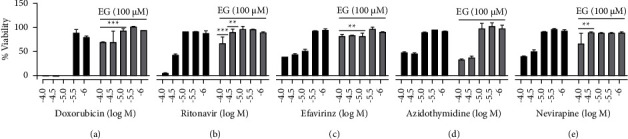
Cytoprotective effect of EG (100 *μ*M) on ART toxicities exerted in U87 MG cells. (a) Doxorubicin (assay control); (b) ritonavir; (c) efavirinz; (d) AZT; (e) nivirapine. Statistical analysis is performed by the paired *t*-test, ^*∗*^shows *p* value <0.05, ^*∗∗*^shows *p* value <0.01, and ^*∗∗∗*^shows *p* value <0.001.

**Figure 8:**
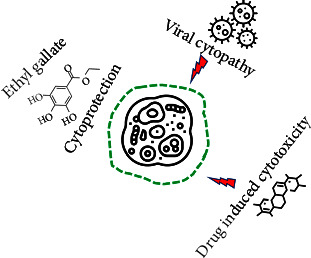
Summary of EG cytoprotection against viral-induced cytopathy and drug-induced cytotoxicity.

The cytotoxicity effect of HIV drugs on liver cells (THLE-3) was rescued by using EG and results are illustrated in Figure 6. Cell viability of HT-29 was prominently decreased by more than 70% when exposed to ritonavir, efavirenz, and nevirapine at 30 *μ*M and above, but AZT was nontoxic up to the concentration of 100 *μ*M. The cell death caused by HIV drugs was effectively abrogated by EG (100 *μ*M), and this salvage effect was more profound at higher doses of allied cell death. Outwardly, the usage of 100 *μ*M EG was able to diminish the liver cell toxicity exerted by the HIV drugs. Ritonavir has been showed many folds of toxicity mainly severe hepatotoxicity resulting in higher chances of hyperbilirubinemia [24]. Supplementing EG with these hepatotoxic drugs could be beneficial to minimize the adverse effects. Among the four HIV drugs tested on brain cells (U87-MG), ritonavir exhibited severe toxicity at higher concentrations followed by other drugs efavirenz, nevirapine, and AZT. Likewise, in other cells where cytotoxicity was induced by HIV drugs, EG effectively showed protection from drug and persuaded toxicity in microglial cells (Figure 7). Overall, EG showed cytoprotection effect not only in HIV-1-induced cytopathic conditions but also rescued the cells from the toxicity of different ART drugs (Figure 8).

## 4. Discussion

The range of HIV drug lethality is comprehensive, comprising gastrointestinal symptoms, renal toxicity, metabolic and cardiovascular complications, skin allergy, weight gain, neuropsychiatric signs, and hypersensitivity [25]. The four HIV drugs tested in the present study are well-reviewed in various clinical settings or stages. In general, older antiretroviral medications such as AZT exhibit serious side effects such as serious oxidative stress and myopathy [26]. Chen et al. [27] demonstrated that EG efficiently inhibited H_2_O_2_-induced cytotoxicity and diminished ROS levels. Recently, Rohilla et al. [28] reviewed the importance of cytoprotective agents, which have to be developed to minimize the toxicity linked with chemotherapeutic agents. Efavirenz is one of the strong NNRTI drugs but discontinued in antiretroviral regimens mainly due to neuropsychiatric and hepatic illness; in some cases, it leads to hepatic failure and death [29]. In the same way, nevirapine also deliberates a risk of severe adverse effects like hepatotoxicity [30]. The main symptoms connected with ritonavir comprise many gastrointestinal issues like vomiting, diarrhea, nausea, and abdominal pain. These outcomes are serious with higher doses of ritonavir. Though extremely toxic antiretroviral medications such as efavirenz are gradually withdrawing from the marketplace, additional research is quite desirable to fully differentiate ART toxicity and its effect to comorbidities witnessed in HIV patients, particularly in the setting of aging, which changes both drug kinetics and vulnerability of the host to ART-related adverse effects [31]. The major adverse impact of zidovudine (AZT) on long-term use caused precisely anemia and neutropenia, which raised 16% and 24% of patients, correspondingly [32]. Overall, cytoprotection against oxidative and chemo toxicities can be achieved either by direct or indirect antioxidants which trigger cellular pathways such as Keap1/Nrf2/ARE, sequentially upregulating the cytoprotective proteins [33].

## 5. Conclusion

Antioxidants are known to protect cells from different toxicities. In this context of HIV drug toxicity, we tested an antioxidant, EG, to rescue the cells not only from the HIV-1 cytopathic effect but also from ARV-associated cytotoxicity. EG effectively inhibited the HIV-1-induced cytopathic effects like syncytia formation and retained the cells to their normal phenotype and also protected them from viral allied cell death. At higher concentrations, the HIV drugs caused more cell death in different cell lines; EG at 100 *μ*M protected the cells from this drug-related toxicity along with their enormous rescue properties against HIV-1 infection. Therefore, from our investigation, we can conclude that EG undoubtedly restored the cell viability from the toxic doses of HIV drugs and it could be a protective supplement along with its potential antiviral and antioxidant benefits.

## Data Availability

The data used to support the findings of this study are available from the corresponding author upon request.
